# Facial Motion Analysis beyond Emotional Expressions

**DOI:** 10.3390/s22103839

**Published:** 2022-05-19

**Authors:** Manuel Porta-Lorenzo, Manuel Vázquez-Enríquez, Ania Pérez-Pérez, José Luis Alba-Castro, Laura Docío-Fernández

**Affiliations:** atlanTTic Research Center, University of Vigo, 36310 Vigo, Spain; mporta@gts.uvigo.es (M.P.-L.); mvazquez@gts.uvigo.es (M.V.-E.); aperez@gts.uvigo.es (A.P.-P.); ldocio@gts.uvigo.es (L.D.-F.)

**Keywords:** facial expression recognition, facial landmarks, action units, convolutional neural networks, graph convolutional networks

## Abstract

Facial motion analysis is a research field with many practical applications, and has been strongly developed in the last years. However, most effort has been focused on the recognition of basic facial expressions of emotion and neglects the analysis of facial motions related to non-verbal communication signals. This paper focuses on the classification of facial expressions that are of the utmost importance in sign languages (Grammatical Facial Expressions) but also present in expressive spoken language. We have collected a dataset of Spanish Sign Language sentences and extracted the intervals for three types of Grammatical Facial Expressions: negation, closed queries and open queries. A study of several deep learning models using different input features on the collected dataset (LSE_GFE) and an external dataset (BUHMAP) shows that GFEs can be learned reliably with Graph Convolutional Networks simply fed with face landmarks.

## 1. Introduction

Facial expressions are one of the most valuable signals in human interactions. Numerous studies have been conducted on automatic Facial Expression Recognition (FER) due to its practical importance in human–robot interaction, personalized medical treatment, driver fatigue monitoring, customer behavior, etc. In the 1970s, Ekman and Friesen [1] postulated that six basic facial expressions of emotion are perceived by humans in the same way regardless of their culture. Today, these facial expressions are the basis of most computer vision systems for FER. However, these facial expressions of emotion are not sufficient to fully represent the expressiveness of human affection, emotion and communication. Human communication uses nonverbal channels to fully convey a message and its context, and facial expressiveness is one of the most important nonverbal channels. In this context, facial expressions are referred to as Linguistic or Grammatical Facial Expressions (GFEs) [2], as they serve a grammatical function in the sentences. An extreme case of the importance of GFEs is that of sign languages, where they provide adjectivation and can modify the semantics of signs [3]. As shown in [4], many studies have been conducted to recognise basic facial expressions of emotion using a wide variety of input features and classification methods. While static images provide enough information to decently perform such a task [5], more subtle facial expression cues require temporal information [6]. Several large datasets for FER are available both with static and dynamic information. Unfortunately, very few datasets containing video representations of grammatical facial expressions are available, making the development of robust models for GFER extremely difficult. Due to such lack of labelled data, using RGB sequences for automated GFE recognition yields to model overfitting the data. Hence, it is good practice to use previously trained feature extractors for related tasks.

In 1978, Paul Ekman created a taxonomy of facial expressions, the Facial Action Coding System (FACS, [7]). From this system, a set of atomic facial muscles (AUs) is defined, from which any further complex expression or emotional state can be inferred. AUs span a large number of combinations that can be used for the recognition of the 6 basic emotions (happiness, surprise, anger, fear, sadness and disgust), as well as for other complex psychological states, such as depression or pain [8]. AUs have also been studied in combination with vocal prosody, as in the series of AudioVisual Emotion Challenge AVEC2016 [9], highlighting their link with non-verbal communication.

Two of the most used features pre-computed for facial expression recognition are Action Units (AUs) [10] as well as facial landmarks [11] that convey discrete information on head and face muscle movements. Techniques to extract both types of features have been greatly improved in the last years thanks to deep models trained with large datasets [4]. These features have been used to perform FER with standard data classifiers as Support Vector Machines (SVMs) [12] or Multilayer Perceptrons (MLPs) [13], or used as complementary input to derive more complex representations using deep neural networks [14].

In this work we try to push the state of the art on grammatical facial expression recognition systems fed with non-RGB data, namely Action Units or facial landmarks, casting these inputs as graphs. Recently, Graph Neural Networks (GNN) and their convolutional extension to Graph Convolutional Networks (GCN) [15] stand out for their flexibility and their good performance in Human Action Recognition. Furthermore, some works have already combined GNNs and facial landmarks [16] as well as AUs [17], obtaining SOTA results in FER.

The main contributions of this work can be summarized as follows:

- A new dataset specifically acquired for GFE in the context of sign languages providing their face landmarks and AUs.
- A thorough assessment of GCN for Grammatical Facial Expression Recognition (GFER) with two types of input features (facial landmarks and action units) and comparison of this technique over two different GFE datasets and also against classical CNN techniques.
- A comparison of the experimental results with human performance on the new dataset.

The rest of the paper is organized as follows: Section 2 reviews the most recent approaches related to the automated recognition of FER/GFER using facial features extracted from video sequences. Section 3 describes the selected datasets and feature extraction, sampling and pre-processing. Section 4, describes the deep learning models and evaluation metrics. Section 5 presents the experiments performed, and finally, Section 6 draws some conclusions.

## 2. Related Work

Our research is focused on communicative facial expressions related to sign languages. As pointed out in [2], signers must be able to quickly identify and discriminate between different linguistic and affective facial expressions in order to process and interpret signed sentences. Through fMRI studies, they demonstrated that the parts of the brain that are activated when detecting emotions and language-related facial expressions are different. It is clear then, that head and facial muscle movement in this context could have common features with facial expressions of emotion, but also their own specificities that should be learned from sufficient input samples.

Many more machine learning studies have been conducted on FER than on GFER, so taking advantage of the techniques developed for FER should be a priority when addressing GFER, especially considering that, as presented in the next section, the number of databases prepared for automated GFE analysis is very small. Thus, we analyzed the research articles on FER and GFER available in the SCOPUS database on the basis of the primary keywords indicated in the Table 1. All searches were restricted to journal and conference articles published between 2009 and 2021 (to cover mainly the trend of deep learning approaches), in Computer Science, Engineering and Mathematics subject areas. This search approach retrieved a total of 929 documents. After a review of titles, abstracts and keywords, it was clear that the vast majority was related to FER using RGB video input, CNN-based approaches, detection of Action Units and, in a lesser extent, landmarks detection. As we were primarily interested in non-RGB inputs to avoid the necessity of large datasets, and to include graph-based systems, we run a secondary search, applying an exclusion criteria based on the secondary keywords given in Table 1. This filtering resulted in a total of 120 relevant documents, again most of them related to CNNs. A final filtering including the term “graph convolutional networks” yield nine relevant studies which where analyzed in depth.

Existing deep learning approaches for video-based FER from facial landmarks, typically concatenate their coordinates over multiple frames to form a sequence of vectors as the input to Recurrent Neural Networks (RNNs) [18], or rearrange them to form an image-like matrix to feed a Convolutional Neural Networks (CNNs) [14]. As comparative results showed, these methods are not able to fully capture the joint dynamics of spatial and temporal features encoded in a sequence of facial landmarks. In the last years, many of the approaches proposed for human action recognition (HAR) use the estimation of body skeleton keypoints. In order to capture the complex spatial-temporal characteristics of human actions, the best performing approaches plug these keypoints, or a transformation thereof, as features of nodes in a graph convolutional neural network, such as Spatio–Temporal Graph Convolutional Networks (ST-GCNs) [19]. Recently, a GCN-based method has been proposed in [16] which uses only facial landmarks for FER. They showed SOTA performance on three large datasets and better performance when fusing with RGB-based models, highlighting the complementary of the approaches when enough data is available. Also, in [20] the Progressive Spatio–Temporal Bilinear Network (PST-BLN) method was proposed for compact modeling of facial expression recognition. They showed performance only slightly worse than RGB based models over three large FER datasets but with a model one order of magnitude smaller. Node features different to landmarks have been also explored. In [17] nodes are defined in a face graph with features related to AUs and edges related to landmark distances. The solution compares favourably against other FER methods over the same large datasets.

These SOTA methods show that FER can be reliably attained with GCN models that do not use RGB information explicitly, which allows leveraging facial feature extraction techniques from large RGB FER datasets. As we will show in the next section, datasets for GFER are really scarce and small, so GCN based approaches over landmarks and AUs are a promising approach.

## 3. Materials

In this section the details of the datasets are described, including the extraction and pre-processing of both action units and facial landmarks and the sampling and normalization of videos.

### 3.1. Datasets

One of the biggest problems in GFER is the lack of extensive and properly collected data. Table 2 summarizes the main datasets collected for dynamic facial expression recognition sorted by number of citations.

Most of the datasets were acquired for FER. In fact most of the works reviewed in our study have used the four first datasets in the table. From those that had different annotations to the basic six emotions + neutral, FABO, LILiR, SILFA, BUHMAP, and GFE-LIBRAS, only the last three were particularly acquired for Sign Language studies. Unfortunately, GFE-LIBRAS, that has a large number of sign language face motions and expressions (9), comprises only 18 clips from 2 persons and only the landmarks are available for download, SILFA is a quite interesting GFE dataset that can be obtained from the authors but it is annotated with a set of AUs, instead of comprehensible sign-language expressions. On the other hand, BUHMAP contains five sign-language-related expressions + two emotion + neutral and the 440 videos from 11 persons are available in RGB videos, so we chose this dataset to test the approaches developed in this work.

#### 3.1.1. BUHMAP

The Boğaziçi University Head Motion Analysis Project Database (BUHMAP) [28] contains labelled videos of 8 different classes of facial signs. The dataset consists of 440 videos recorded by 11 different subjects (6 women and 5 men) which performed each sign 5 times. The included classes combine basic emotions with head movements acted by the donors when prompted. The classes are defined below:

1. *Neutral*: The neutral state of the face. The subject neither moves his/her face nor makes any facial expressions.
2. *Head L-R*: Shaking the head to right and left sides. The initial side varies among subjects, and the shaking continues about 3–5 times. This sign is frequently used for negation in Turkish Sign Language (TSL).
3. *Head Up*: Raise the head upwards while simultaneously raising the eyebrows. This sign is also frequently used for negation in TSL.
4. *Head F*: Head is moved forward accompanied with raised eyebrows. This sign is frequently used to change the sentence to question form in TSL.
5. *Sadness*: Lips turned down, eyebrows down. It is used to show sadness, e.g., when apologizing. Henceme subjects also move their head downwards.
6. *Head U-D*: Nodding head up and down continuously. Frequently used for agreement.
7. *Happiness*: Lips turned up. Subject smiles.
8. *Happy U-D*: Head U-D + Happiness. The preceding two classes are performed together. It is introduced to be a challenge for the classifier in successfully distinguishing this confusing class with the two preceding ones.

**Figure 1:**
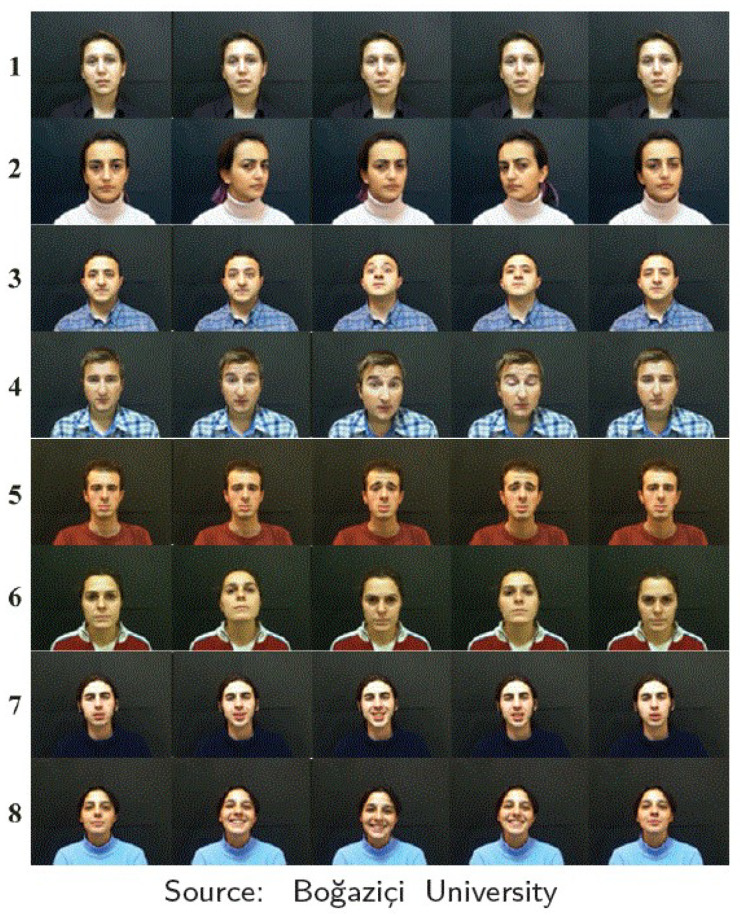
Example of FE classes in BUHMAP dataset. Rows from top to bottom: 1—Neutral, 2—Head L-R, 3—Head Up, 4—Head F, 5—Sadness, 6—Head U-D, 7—Happiness, 8—Happy U-D.

Figure 1 shows an example of frame sequences for the 8 defined classes in BUHMAP dataset.

#### 3.1.2. LSE_GFE

LSE_GFE has been extracted from the LSE_UVIGO [33], a multi-source database designed to foster research on Spanish Sign Language Recognition. The anonymous data needed to reproduce the experiments have been released, jointly with all the code, in the github page https://github.com/mporta-gtm/GrammaticalFacialExpressions (accessed on 1 May 2022). The dataset contains isolated signs, expressive sentences and interviews, all acquired in a controlled lab environment. Also, besides the sign/gloss/sentence labels, 841 videos have also annotations for some grammatical facial expressions, namely:

1. *q.polar*: Yes/no question. Head and body is slightly moved forward accompanied with raising eyebrows. This sign is frequently used to change the sentence to close question form in LSE. 123 samples performed by 19 people.
2. *q.partial*: Open question. Head and body is moved forward accompanied with frown eyebrows. This sign is frequently used to change the sentence to open question form in LSE. 265 samples performed by 13 people.
3. *q.other*: General question form, not assimilable to polar (close) or partial (open). 16 samples from 9 people.
4. *n.L-R*: Typical “no” negation, similar to Head L-R in BUHMAP. 176 samples performed by 22 people.
5. *n.other*: General negation, not assimilable to n.L-R. 53 samples from 19 people.
6. *None*: Samples without any of these questioning or negation components were extracted from the available videos of 24 people, to ensure the capacity of the model to detect the presence of non manual components. This class is quite different to the BUHMAP *Neutral* class, because in the LSE_GFE case, other communicative expressions can be included in the *None* class, i.e, dubitation with complex head and eyebrows movement.

The statistics of this dataset are shown in Table 3. It can be seen that the data distribution is highly unbalanced both in gender and in classes. This problem would be tackled in future research.

This dataset had to be filtered before using it in order to solve some detected issues. First, as classes *q.other* and *n.other* had too few samples and their definition was ambiguous even for expert language interpreters, they were discarded. Second, not all the collaborators which recorded the videos were deaf people so, to maintain the integrity of the dataset, ensuring that all samples were correctly performed, only recordings from deaf people and sign language interpreters were considered. The final number of videos for the dataset in this work was 413, that compares to the 440 videos of BUHMAP.

**Figure 2:**
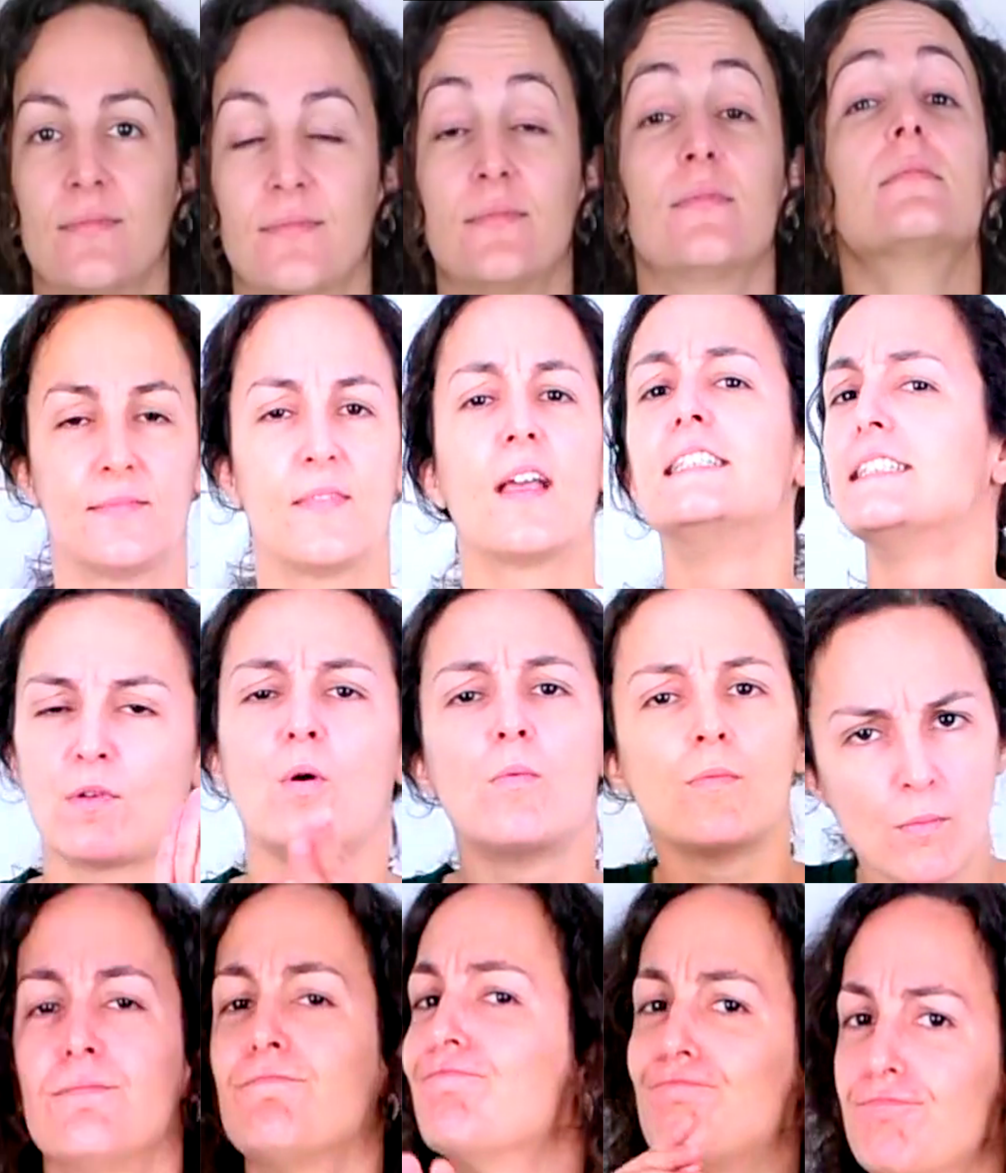
Example of FE classes in LSE_GFE dataset. Rows from top to bottom: None, q.polar, q.partial, n.L-R.

Figure 2 shows an example of frame sequences for the 4 defined classes of LSE_GFE for this work.

**Figure 3:**
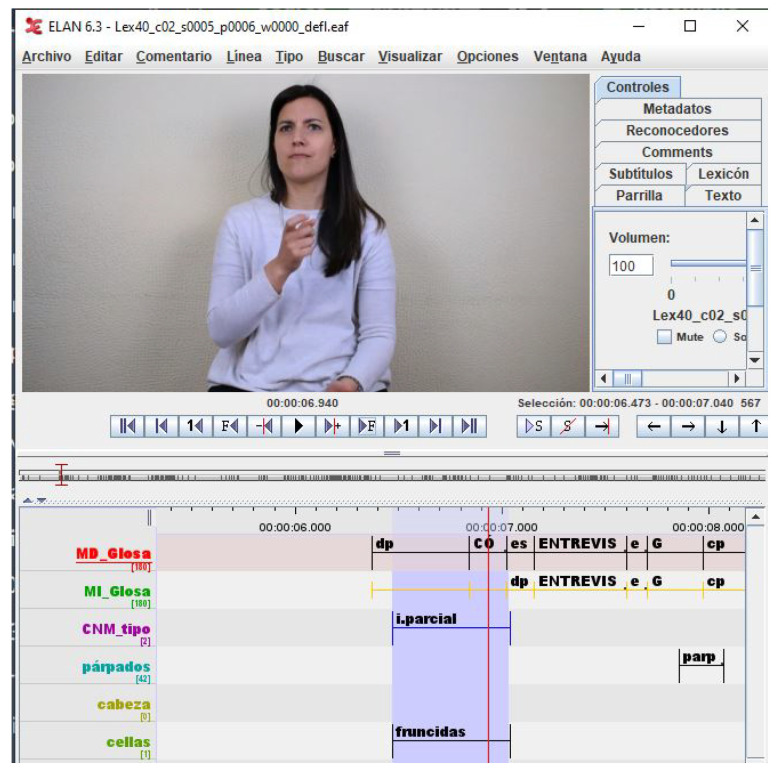
Snapshot of the ELAN annotation tool with an example of interval of an open question class *q.partial*.

It is important to note that the GFEs of LSE_GFE are extracted from interviews, so facial expressions are expected to be more natural than in the case of BUHMAP, where the 8 classes were forcibly generated. Figure 3 shows a snapshot of the ELAN tool [34] used for annotating the Lex40_UVIGO dataset. In this snapshot an example of annotation of an interval for the class *q.partial* (i.parcial, in spanish) can be observed.

### 3.2. Features

#### 3.2.1. Action Units

**Figure 4:**
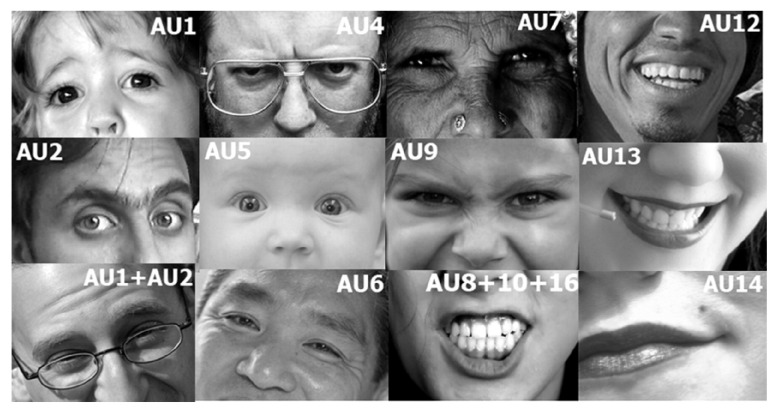
Examples of Action Units. Henceurce: OpenFace.

Action Units are defined as the fundamental movements of muscles or groups of muscles of the face that correspond to the display of an emotion. These movements are encoded by the Facial Action Coding System (FACS) [7]. Using FACS it is possible to code most anatomically plausible facial expressions by disassembling them into specific Actions Units (AUs). An example of some action units is supplied in Figure 4, where the activation of groups of muscles is labelled with the corresponding AU code.

The extraction of Action Units was carried out using OpenFace [35], a face analysis library that includes a state-of-the-art HOG-based method for detecting AUs. This model provides the presence (binary value) of 18 AUs together with an estimation of the intensity value, from 0 to five, of 17 of them. This data was pre-processed to build a vector of 18 values were the first 17 are the obtained intensities and the last one is the presence value of the remaining AU scaled to the range of the intensities. Then, these vector were concatenated in a matrix where the horizontal axis contains the 18 AUs studied and the vertical axis represents their temporal evolution.

#### 3.2.2. Facial Landmarks

Facial landmarks were extracted also by using OpenFace, which employs a deep learning state-of-the-art method [36]. After the extraction, the x and y coordinates of 68 keypoints are normalized between −1 and 1 with respect to the landmark of the nose tip, where −1 and 1 correspond to the points furthest away from the nose. In addition, in some experiments a data augmentation technique consisting of horizontal flipping of x landmarks coordinates was tested. Finally, the use of 3D coordinates was considered but preliminary tests show that the depth estimation of this method is noisy and does not contribute to enhance the performance on the evaluated models.

### 3.3. Video Sample Generation and Preprocessing

**Figure 5:**
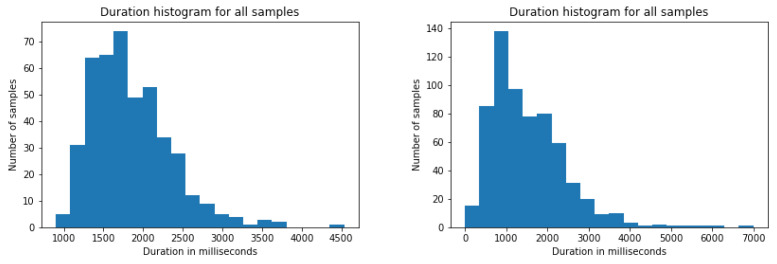
Duration of annotated segments in BUHMAP (**left**) and LSE_GFE (**right**).

The assessed classification models require the input samples to have an equal, or at least very similar, size. In order to gain insight of the labelled events of each dataset a small study on their duration was carried out. Figure 5 shows the distribution of such events in both datasets in terms of duration in milliseconds. It can be seen that both datasets follow a non-gaussian distribution with different means and deviations (1.5 s ± 0.9 for LSE_GFE and 1.8 s ± 0.5 for BUHMAP). Furthermore, the longest event in LSE_GFE last 7 s while BUHMAP videos have a maximum length of 4.5 s. These differences talk about the different acquisition setting of both datasets: in BUHMAP volunteers perform a prompted movement/expression, in LSE_UVIGO volunteers respond naturally to questions asked by a deaf interpreter in sign language. In addition, the variation on acquisition frame rate between both sets is also rather significant, as BUHMAP videos were recorded at 30 frames per second while LSE_UVIGO has recordings at 50 fps (38% of the total) and 60 fps (the remaining 62%). Due to all these differences, it is reasonable to conclude that the size of the input samples from both datasets will probably have to be different in order to best fit the kind of events that it pretends to explain. The duration and frame rate were also included in the study.

**Figure 6:**
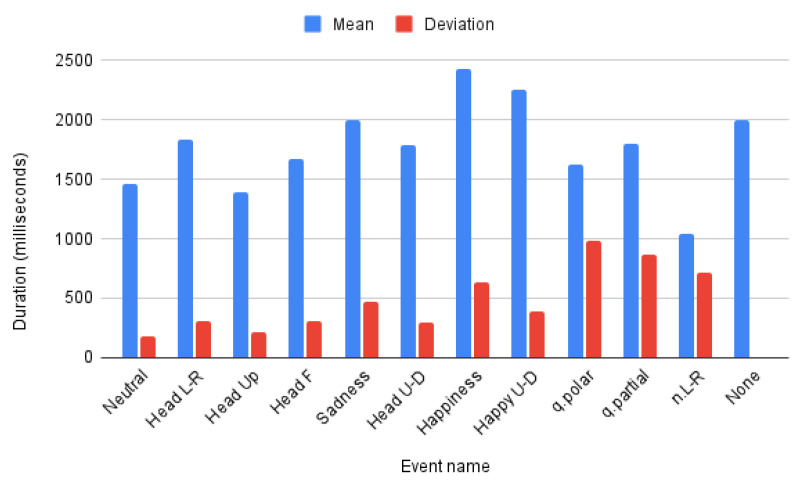
Duration per class in LSE_GFE and BUHMAP.

In addition to the inter-datasets length differences there also exist inter-class differences for both datasets. Mean and deviation of each class lengths are presented in Figure 6. It is worth noting that the *None* class of LSE_GFE dataset has no deviation as its samples are obtained as fixed size sequences without target events and might contain any other emotional FE or GFE not being studied in this work, so it is not, in general a neutral expression. Furthermore, the classes in LSE_GFE have a higher deviation and particularly class *n.L-R* has a shorter mean duration, which can influence the obtained results.

Video samples were generated by cropping a window with the selected duration centered in each labelled event of each video. An ablation study includes the effect of cropping duration and frame-rate.

## 4. Methods

This section covers the definition of classification models and the evaluation methods.

### 4.1. CNN Models

In order to verify the effectiveness of GCNs for GFEs recognition three different CNN architectures are assessed: the *VGG*, model the *MobilenetV2* [37] model and a custom CNN with several convolutional depths.

**Figure 7:**
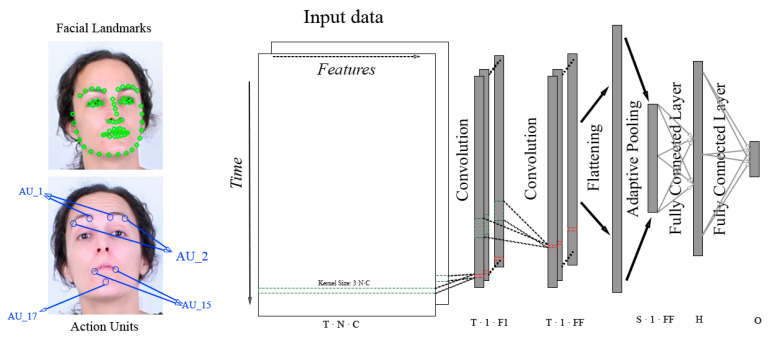
Overview of custom CNN architecture. The input data, whether facial landmarks or action units, is arranged in a matrix of shape T(temporal_length)×N(number_of_features)×C(dimensionality_of_features). This data is processed using F1 convolutional filters of shape 3×N×C to obtain F1 feature vectors of shape T×1. Then, extra convolutional blocks with different number of filters can be added resulting in a final set of FF feature vectors with shape T×1. After that, these feature vectors are flattened and an adaptive pooling reduces their temporal dimension to *S*, obtaining a set of feature vectors with shape S×1×FF. Finally, those vectors are processed using two fully connected layers with a hidden space of *H* neurons resulting in *O* output values.

The rationale behind building a custom CNN instead of just using of-the-shelf models was to accommodate the kernels to the non-image nature of the input. As commented in Section 2, feeding a CNN with sequence of landmarks can be done by arranging them as concatenated rows in time [14], forming a 1-channel image if X-Y(-Z) coordinates are concatenated, or a 2(3)-channels X,Y(,Z). Once this artificial image is built one can use classical CNNs with typical square kernels, like VGG or mobilenet, or rectangular kernels that span one dimension along the length of the feature vector. We have adopted the latter for the custom CNN, both for landmarks and for AU features. Therefore, the custom CNN was composed using a convolutional block with 64 filters of size (5, *size of input features*) and (2, 0) padding, compacting the feature dimension to a single value for each frame while maintaining the temporal resolution. Regardless, more convolutional blocks can be appended to increase the model depth as shown in Figure 7. After an adaptive max pooling, the resulting 64 vectors are flattened and classified using two fully connected layers with a hidden space of 128 neurons. Furthermore, batch normalization is applied after the convolution and a ReLu activation follows the convolution and the first linear layer, while the output of the second linear layer is activated using a softmax function. This model has only ∼30 k parameters when only one convolutional block is used.

### 4.2. GCN Models

GNNs are designed to work with not regularly sorted data and can broadly be classified into two classes: spectral and spatial GNNs. The main difference between them is that spectral GNNs convolve the input graph with a set of learned filters in the graph Fourier domain while spatial GNNs, in general, perform layer-wise updates for each node by, first, selecting neighbors, then merging the features from the selected neighbors with an aggregation function and finally applying a transformation to the merged features. Graph Convolutional Networks (GCNs) are considered to be a spatial GNN variant characterized to perform mean neighborhood aggregation through convolution operations. This networks can be seen as a generalized version of Convolutional Neural Networks (CNNs) in which the data do not need to follow an order and the number and distribution of neighbouring nodes can vary, since the neighbourhoods are not based on spatial constraints but on defined relationships between nodes. To do so, a new element is added in the forward step Equation (1). In such equation the weights $W^{i}$ of the i-th convolutional filter multiply the input features (nodes) $X^{i}$ and the adjacency matrix (edges) *A*, which represents the relationship between the input features. This matrix has shape N×N, where *N* is the number of input nodes.

(1) $$H^{i+1}=\sigma \left(W^{i}X^{i}A\right)$$

**Figure 8:**
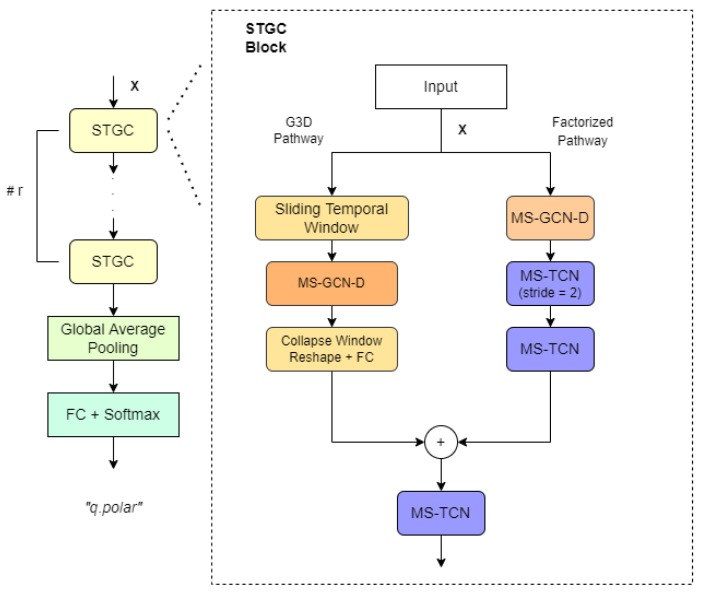
Overview of MSG-3D architecture. “TCN” and “GCN” denote temporal and graph convolutional blocks, and prefix “MS-” and suffix “-D” denote multi-scale and disentangled aggregation.

The GCN selected for this work is based on a recent state-of-the-art model with great success in action recognition [38] (onwards, *msg3d*) and also in sign language recognition [39]. The representational capacity of this spatial-temporal model in HAR and SLR moved us to try testing it for GFER. If the model is able to capture GFE then we can hypothesize that a unified body skeleton and face mesh might be able to find the linguistic relationship between manual and non-manual (including GFE) components in sign languages. This model was adapted in this work to fit both facial landmarks and action units, matching each face landmark or action unit to a node feature of the graph. The structure of the full model is depicted in Figure 8. In short, it stacks *r* (3 in our case) spatial-temporal graph convolutional (STGC) blocks to process input features and then applies an average pooling and a softmax classifier on a fully connected layer.

One of the main advantages of this model is the use of multi-scale disentangled graph convolutions. The goal of this operation is to take into account connections between nodes which are separated by several hops. Previous proposals [40] employed higher-order polynomials of the adjacency matrix to aggregate multi-scale structural information but this method suffers of a bias towards local regions as, even though it takes into account longer paths between nodes, the amount of shorter paths outweigh them. In order to avoid this problem, the authors build the *k*-adjacency matrix (where *k* stands for the number of scales used, i.e., the maximum path length between two nodes that is taken into account) as

(2) $$A_{\left(i,j\right)}^{k}=\left\{\begin{array}{cc} 1 & \mathrm{if}\;d(v_{i},v_{j})=k\\ 1 & \mathrm{if}\;i=j\\ 0 & \mathrm{otherwise} \end{array}\right.$$

where $d(v_{i},v_{j})$ gives the shortest distance in number of hops between nodes $v_{i}$ and $v_{j}$.

STGC blocks deploy two paths in order to extract regional as well as long-range spatial and temporal correlations: The first path (called G3D pathway) uses sliding spatial-temporal windows to sample small regions, performs disentangled multi-scale graph convolutions on them and collapses them with a linear layer. On the other hand, the second path (called factorized pathway) chains three different layers to obtain long-range, spatial-only and temporal-only information. The first layer performs disentangled multi-scale graph convolution only in the spatial dimension with the maximum number of graph scales, obtaining a long-range representation of spatial information for each temporal unit. Then, in second and third layers, it performs multi-scale temporal convolutions over the result of the first layer, thus capturing extended temporal information.

The model also includes a trainable mask which is added to the adjacency matrix *A* before each convolution step, allowing the network to learn directly the best connections between nodes. Unfortunately, the use of multiple scales and temporal windows scale up the size of this mask to tens of thousands of trainable parameters.

In our, pre-computed feature sequences were re-structured to build a graph, using each landmark coordinate or action unit intensity value as a node feature and defining relationships between all nodes. As the proposed method takes into account both spatial and temporal information in a unified model, the input to the model has to be a sequence of feature graphs corresponding to consecutive frames.

**Figure 9:**
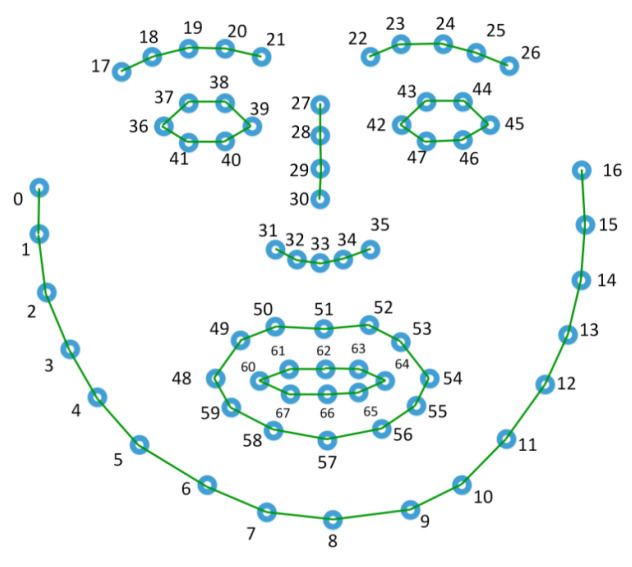
Base graph used with MSG3D.

In order to test the influence of the input connections, particularly when using facial landmarks as input, two different graphs were defined and used in this work. The first one, named *base graph* and shown in Figure 9, define connections between the key-points with stronger muscle or anatomical relation. The second graph does not define any connection and rely on the ability of the model to learn relevant connections from scratch. In the case of action units, a single fully-connected graph was used in which all nodes were connected to each other.

Finally, an ablation test was carried out to study the impact of the number of trainable parameters of the model in the obtained results. To do so, the same tests were replicated using a single scale in both spatial and temporal dimensions.

### 4.3. Evaluation Strategy and Metrics

To evaluate the performance of each configuration Leave-One-Subject-Out (LOSO) cross-validation was used. This method divides the dataset in as many folds as different persons participate in it. Then, the model is trained from scratch once for each division, using the selected fold as validation data and all the remaining folds as training data.

Regardless, as the LSE_GFE has a large number of collaborators which contributed with different number of samples non-uniformly distributed over the classes, only the 11 persons with more and best distributed samples were used. Remember that BUHMAP is composed by 11 persons. The ids of the selected individuals as well as the number of samples from each class they recorded are depicted in Table 4.

In addition, to tackle the randomness of stochastic gradient descent, which resulted in substantial differences between reiterated trainings of the same model, each evaluation needed to be repeated ten times to extract a reliable estimation of the model performance. Mean and ± standard deviation are provided for every test.

The metrics selected to evaluate the performance of the model are F1 score, for its capacity to assess precision and recall in a single value, and the accuracy. Finally, as each fold could contain a very different number of samples per class and the individual metrics of each split need to be weighted in order to achieve a reliable mean, the output of the model over the validation set of each fold was stored and concatenated in a single matrix. Then, the metrics are computed directly from this matrix, obtaining the equivalent to a weighted mean of all the folds. In addition, as the F1-score is defined for binary classification, it is computed individually for each class versus all others and then the weighted mean among all classes is obtained, taking into account the number of samples of each one in the validation set.

## 5. Results

In this section, the results obtained for all the relevant experiments carried out are presented and discussed.

**Comparative study of classical CNN models.**

In this study we compared the performance of VGG-11 and MobilenetV2, as examples of deep networks with different convolutional blocks, with the much smaller custom CNN model presented above. All of them were trained from scratch because there are not pretrained models for these type of inputs. It can be observed from Table 5 that VGG-11 and MobilenetV2 perform worse than the smaller custom CNN model for both datasets and both input features. Deeper custom CNN models (not presented in the Table to avoid overcrowding it) performed worse than the simplest one. It can be argued that VGG-11 and MobileNetV2 were not adapted to the type of input feature and also that the model is too large for the size of the dataset and overfits the data. It is worth noting that allowing a global combination of input features in custom CNN was more beneficial to landmarks than AUs, as the difference is coherent in both datasets. However, the classical local input filters of VGG-11 and MobilenetV2 yields not conclusive results on the advantage of landmarks over AUs, as they depend on the dataset.

**Assessment of MSG3D performance**.

The next set of experiments are focused on assessing the performance of Multi-Scale Convolutional Spatial-Temporal Graph Neural Networks when applied to classifying GFEs. The rationale to bring to this work a network that has proven efficiency for action recognition with skeletal-type inputs might be questionable. Facial expressions do not show the same spatial-temporal variability as the full skeleton of a person in action, but given that GCNs can be seen as a generalization of classical CNNs and there are many model meta-parameters that can be adjusted, a rigorous study might throw interesting results.

Table 6 shows the performance of the baseline MSG3D model for both datasets and input features. It is interesting to highlight that MSG3D outperforms the custom CNN in both datasets when using landmark coordinates as the feature of the GCN nodes. This behaviour is even more interesting when looking at the number of free parameters of the baseline model, which is near three times the size of MobilenetV2. This means that MSG3D is a model much more appropriate to explain these kind of spatial–temporal data than MobilenetV2. Comparing with the lighter custom CNN the performance advantage is not so clear. It decreases a bit for BUHMAP but increases largely for LSE_GFE. However the size of the model is more than two orders of magnitude larger. On the other hand, when the features of the nodes are AUs, performance improves for BUHMAP but decrease dramatically for LSE_GFE. The size of the model is three times smaller than using landmarks because, differently to classical CNNs, it scales with the number of graph-nodes. The poor behavior of AUs in LSE_GFE remains unexplained, but our main hypothesis is that LSE_GFE is a more difficult dataset to extract reliable AUs as it only contains GFEs and OpenFace is optimized to extract AUs for facial emotional expressions. BUHMAP contains a mix of GFEs and emotional FEs. As we do not have control on the accuracy of the AUs extractor of OpenFace, and landmarks (trained for more scenarios than FEs) outperforms or equals AUs for this problem, we will do the next ablation study just using landmarks.

### 5.1. Ablation Study on MSG3D

The objective of this ablation study consists of assessing the influence of several meta-parameters of MSG3D and input sample composition in the final performance for both datasets. The first three experiments deal with the bias-variance dilemma and the generalization capacity of the model. Hence, we will test a simple data augmentation technique, change the flexibility of the model and reduce the size of the model. The second set of experiments will test the influence of the temporal duration of the training sample that contains the GFE, and the apparent frame-rate seen by the model. In short:

- Use of data augmentation through horizontal flipping.
- The impact of graph topology.
- Number of spatial and temporal scales.
- The impact of GFE duration and frame-rate

**Study on the effect of data augmentation through horizontal flipping**.

Table 7 shows the effect of augmenting the input sample with x-flipped node features. Results clearly show that this simple data augmentation improves generalization so, the following tests will include it.

**Study on the effect of graph topology**.

This study tries to assess if imposing a muscular–anatomical graph from the beginning is better than start from an empty graph. It must be highlighted that the model has the capacity to insert and delete connections in both cases during training. Table 8 shows nothing conclusive as imposing the initial graph is better for LSE_GFE but not for BUHMAP, so we will keep the variable in the next ablation test.

**Study on the effect of number of spatial and temporal scales**.

This study tries to evaluate whether the multiscale approach, which increases complexity and gives extra flexibility to the model to learn spatial-temporal dependencies, is worthwhile for this problem. Results from Table 9 shows that BUHMAP is better explained with a simpler model, but it is not so clear for LSE_GFE. As the complexity of the model is greatly reduced removing spatial and temporal scales (SS, TS) and the difference in LSE_GFE is not statistically significant when using empty-graph, we will keep this model for the last ablation test.

**Study on the effect of GFE duration and FPS**.

The last ablation study is not related to the model itself but to the size and temporal redundancy of the input. We already explained that both datasets have been acquired for different purposes and with different acquisition settings, so there’s not a priori information of the optimal duration and frame-rate for feeding the MSG3D in this context. Table 10 shows the result of halving/doubling the duration of the input event inside the limits of the distribution range of both datasets and subsampling the original frame-rate to reduce redundancy (as LSE_GFE is recorded at 50 and 60 fps a slight interpolation effect might be present in some of the tests). Table 10 shows a larger dependency on the duration for LSE_UVIGO than BUHMAP. This can be explained by the larger deviation per class in the former than the latter (see Figure 6). The best results for LSE_GFE are obtained with intervals of 2 s while for BUHMAP there’s a slight improvement using 4 s. The influence of frame rate is not very important in general but a slight improvement is observed with less redundancy probably related to a minor overfitting risk.

### 5.2. Accuracy Per Class

**Figure 10:**
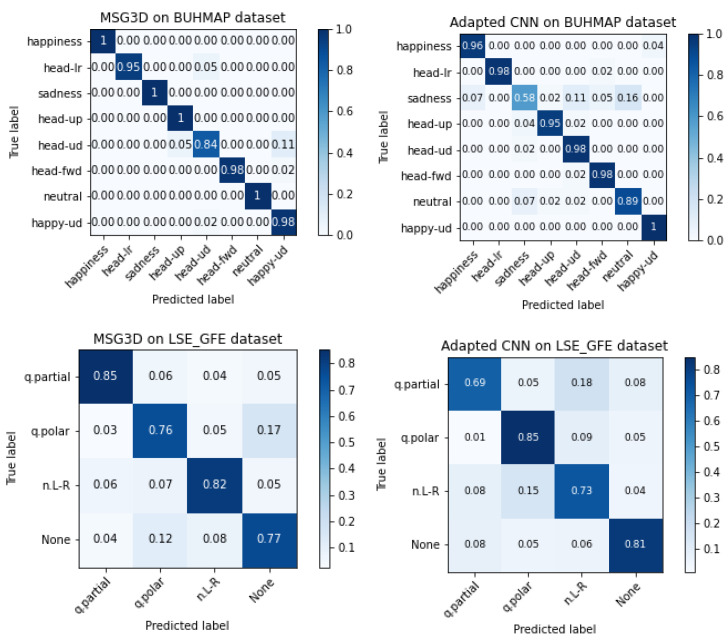
Confusion matrices of MSG3D (**left**) and custom CNN models (**right**) for BUHMAP (**top**) and LSE_GFE (**bottom**).

In order to understand the difficulty of learning each specific class, the confusion matrices of the best models for each dataset and type of model are presented in Figure 10 only for landmark features. It is worth noting that the two architectures present an apparent complementary behavior, as those classes with worse accuracy in MSG3D have better or similar accuracy using the custom CNN, in both datasets. Complementary of models performance can be exploited for fused decisions, but drawing solid conclusions on fused classifiers must be handle carefully when datasets are as small as in this work. This study is left for future research if LSE_GFE is increased.

### 5.3. Comparison with State-of-the-Art Methods on BUHMAP

The papers published using BUHMAP dataset are a little bit old and most of them use different subsets of it, excluding some of the labelled classes. In addition each study uses different test strategies and report different metrics. In [41] the authors use all the eight labelled classes and report the mean test accuracy over LOSO. In [42] the samples of neutral expression are excluded and the reported results are the mean classification accuracy over LOSO. Ref. [43] uses a subset of the database focused on the three classes related to facial expressions and report the classification accuracy and the F1-score but no explanation about the used evaluation data is given. Finally, ref. [44] performs three different tests: The first one uses samples from seven classes (excluding neutral expression class) from one person to train and the same samples from other subject to test. The second one performs five-fold cross-validation over the samples of seven classes from two different persons. The last one uses three repetitions of four classes related to head movement from nine subjects to train and test on the remaining two subjects. The numerical results reported in the publications of these systems are gathered in Table 11 and compared with the best result obtained for the BUHMAP dataset in this work, which was achieved by training a reduced *MSG3D* model over the facial landmarks augmented through horizontal flipping. The only systems that outperform our proposal are not fully comparable as they remove four classes [44] or just the neutral expression [42]. The latter system is a fusion of several subsystems and an ad hoc selection of feature sets after the merger, which could point to some meta-overfitting. It was not our intention to build the best system for classifying over BUHMAP but presenting a new dataset and propose an alternative deep learning approach that handles spatio-temporal graphs to automate the classification of GFE. Testing over BUHMAP was the only way to make sure that our approaches made sense for this problem, and they could achieve SOTA performance on previous similar datasets.

### 5.4. Comparison with Human Performance on LSE_GFE

For the sake of completeness we made a last study on the performance of sign language experts when watching the same video clips extracted for testing the models. The sign language experts were three sign-language interpreters. Two of them had never seen the complete video interview of LSE_UVIGO from where the GFE video clips were extracted. The other interpreter was the same person (also author of this paper) that labeled the LSE_GFE dataset watching the whole sign language sentence. It is important to highlight that at least 12 months passed from the annotation of LSE_GFE to the experiment we are going to explain here, so this interpreter had mostly forgotten the context of the video clips.

A web-based application was prepared for the interpreters to watch a video clip from a random sequence extracted from the LSE_GFE dataset and manually annotate the 4 studied classes. Due to the difficulty of the problem, also alternative responses could be selected from this set:

- q.doubt -> “I am sure that it is question but I do not know whether it is polar or partial”,
- doubt -> “I am not sure whether it is a question or a negation but I am sure it is one of them”,
- not selected -> “I am not sure of any option. Pass”.

**Figure 11:**
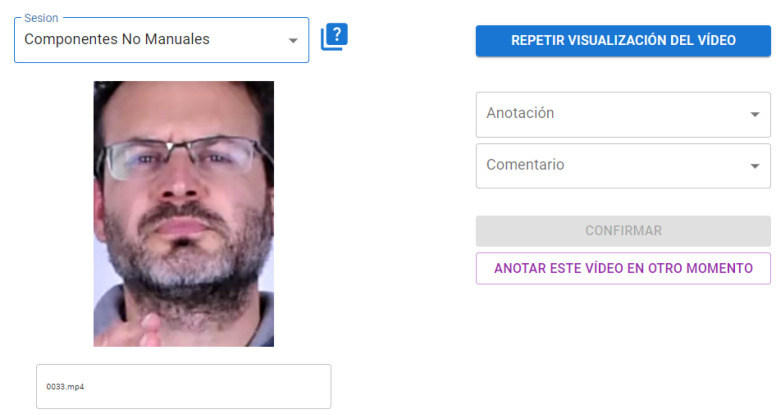
Screenshot of the web tool of LSE_GFE manual annotation.

With these three options we try to minimize the effect of random responses. Figure 11 shows a screenshot of the web tool accessible to the interpreters (in Spanish). They were free to enter in different moments and annotate as in an Amazon Mechanical Turk task. Every annotation was linked to the annotator and deleted from the random list for that annotator.

**Figure 12:**
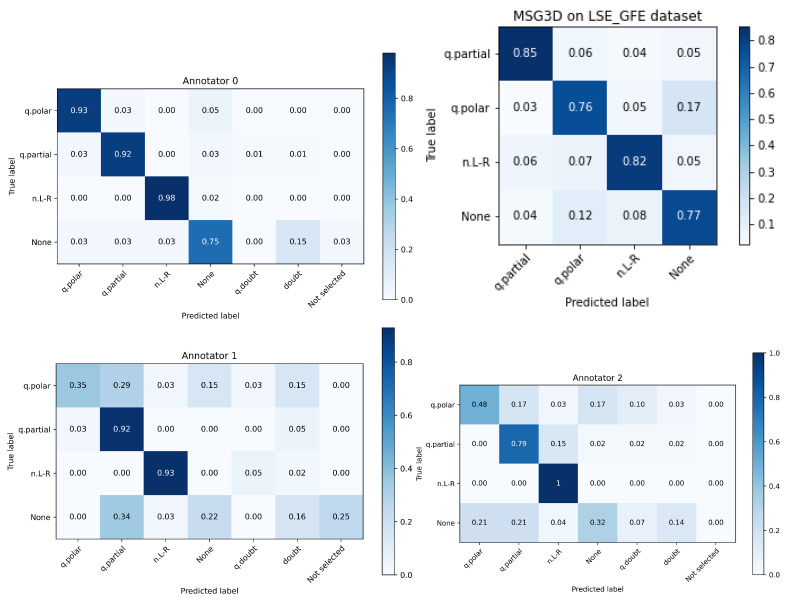
Confusion matrices of the interpreters against the original labels of LSE_GFE. Upper right shows the best automated system.

Figure 12 show the confusion matrices of the different interpreters-annotators. Annotator 0 is the one that 12 months before labeled the dataset watching the whole sign language interviews. The best automated system is again displayed to facilitate comparison with annotators.

Several interesting observations can be drawn from this experiment:

- Annotator 0 performs much better than the others, so it is clear that there’s an influence on having seen the footage and being involved in the acquisition process and discussions on the experiments.
- The class *None*, that was randomly extracted from interview segments where none of the 3 classes were present, is, by a large margin, the class with worse human performance. Even the annotator 0 performed worse than the automated system. This is a clear cue that the larger performance of annotator 0 was boosted by the previous knowledge of the dataset.
- The three annotators outperformed the automated system in classes *q.partial* and *n.L-R*, but two of them performed much worse in class *q.polar* where they showed many doubts.

In summary, this experiment demonstrated that LSE_GFE is a challenging dataset worth distributing to the research community to advance the state of the art in GFE recognition and sign language comprehension. Also, the disagreement among annotators and the advantage of having seen the whole sign language sentence tells us that there is much more work to do regarding the duration of needed context in input features for building a successful automated system.

## 6. Conclusions

This work presented a new dataset of Grammatical Facial Expressions (GFE) acquired in a natural context of Spanish Sign Language (LSE_GFE) and a comparative study of a type of Convolutional Graph Neural Networks for automated classification of GFE classes. This type of GCN has been already successfully applied to FER but, as far as we know this is the first time that this type of complex models, well known in the action recognition arena, are applied to GFE recognition.

In order to assess the model adequacy for the task, all the experiments were carried out with the LSE_GFE and another publicly available dataset, BUHMAP, already gather and studied for FER and GFER in the past. The experiments using landmarks and action units as input features, and a custom CNN and the GCN model called MSG3D, over the two datasets, showed that the best option that surpassed the state of the art was obtained with simplified MSG3D fed with landmarks and augmented data with horizontal coordinate flipping.

Experiments with human expert sign language annotators showed that the simplified MSG3D model is able to compete with them, outperforming class accuracy in two of the four classes. These experiments also showed that the additional temporal context of GFE might be necessary for accurate disambiguation between similar expressive facial movements. Further research on models able to deal with class-dependent input duration could improve accuracy. 

## Figures and Tables

**Table 1:** Bibliographic search keywords.

Primary Keywords	(“facial expression recognition” OR “facial emotion recognition” OR (“linguistic” OR “grammatical”) AND “facial expressions” AND “recognition”) OR (“facial micro-expression recognition”) AND (“video” OR “image sequences” OR “dynamic expressions” OR “temporary information” OR “temporal data” OR “spatial-temporal")
Secondary Keywords	(“action units” OR “landmarks”)
Last filtering	(“action units” OR “landmarks” AND “graph convolutional networks”

**Table 2:** Main datasets collected for dynamic facial expression recognition.

Name	Cites	Type of Expression	Acquisition Set Up	Classes	Videos	Persons
CK+ [21]	1754	emotions	controlled	7	327	118
OULU-CASIA [22]	413	emotions	controlled (light variation)	6	480	80
MMI [23]	342	induced emotions	natural	6	213	30
AFEW [24]	316	emotions	movies	7	1426	330
MUG [25]	261	induced emotions	semi-controlled	6	1462	52
BU-4DFE [26]	154	emotions	controlled	6	606	101
FABO [27]	85	induced emotions	semi-controlled	9	1900	23
BUHMAP [28]	20	sign-language/emotions	controlled	8	440	11
GFE-LIBRAS [29]	10	sign-language	natural	9	36	2
DFEW [30]	8	emotions	movies	7	16,372	-
LILiR [31]	6	non-verbal communication	natural	4	527	2
SILFA [32]	3	sign-language	semi-controlled	?	230	10

**Table 3:** Gender distribution in LSE_GFE dataset (#samples).

Class	Female	Male
*q.polar*	68	55
*q.partial*	174	91
*q.other*	10	6
*n.L-R*	105	71
*n.other*	29	24
*None*	109	99

**Table 4:** Sample distribution (#samples) of LSE_GFE collaborators used in LOSO.

ID	*q.polar*	*q.partial*	*n.L-R*	*None*
p0003	19	47	20	13
p0004	6	4	3	3
p0006	15	49	20	34
p0013	6	15	3	11
p0025	6	14	4	11
p0026	4	12	8	9
p0028	5	18	3	11
p0036	15	30	24	35
p0037	7	18	9	34
p0039	12	19	11	15
p0041	6	13	8	16

**Table 5:** Comparative study of classical CNN models.

Model	Dataset	Feature	Weighted F1	Accuracy	Parameters
MobilenetV2	BUHMAP	landmarks	$75.55{\%}_{\pm 2.78}$	75.11%±2.75	2233768
MobilenetV2	BUHMAP	AUs	$78.30{\%}_{\pm 2.33}$	78.43%±2.26	2233480
MobilenetV2	LSE_GFE	landmarks	$67.16{\%}_{\pm 2.22}$	66.90%±2.24	2228344
MobilenetV2	LSE_GFE	AUs	$62.78{\%}_{\pm 1.12}$	61.43%±1.17	2228356
VGG-11	BUHMAP	landmarks	$77.28{\%}_{\pm 2.64}$	77.50%±2.53	128804040
VGG-11	BUHMAP	AUs	$79.20{\%}_{\pm 2.13}$	79.02%±2.15	128803464
VGG-11	LSE_GFE	landmarks	$66.62{\%}_{\pm 2.27}$	66.89%±2.14	128787652
VGG-11	LSE_GFE	AUs	$63.18{\%}_{\pm 0.99}$	62.21%±1.01	128787076
custom CNN	BUHMAP	landmarks	$88.81{\%}_{\pm 1.30}$	$88.75{\%}_{\pm 1.31}$	31992
custom CNN	BUHMAP	AUs	$85.90{\%}_{\pm 1.21}$	$85.98{\%}_{\pm 1.18}$	23912
custom CNN	LSE_GFE	landmarks	$71.96{\%}_{\pm 1.45}$	$71.89{\%}_{\pm 1.48}$	31732
custom CNN	LSE_GFE	AUs	$70.49{\%}_{\pm 1.00}$	$69.52{\%}_{\pm 1.06}$	23652

**Table 6:** Performance of baseline MSG3D model.

Dataset	Feature	Weighted F1	Accuracy	Parameters
BUHMAP	landmarks	$87.05{\%}_{\pm 1.79}$	$86.75{\%}_{\pm 1.80}$	6527396
BUHMAP	AUs	$87.71{\%}_{\pm 1.48}$	$87.73{\%}_{\pm 1.47}$	2913570
LSE_GFE	landmarks	$79.17{\%}_{\pm 1.45}$	$79.16{\%}_{\pm 1.50}$	6525856
LSE_GFE	AUs	$59.60{\%}_{\pm 0.8}$	$58.24{\%}_{\pm 0.91}$	2912030

**Table 7:** Ablation study for data augmentation through horizontal flipping.

Dataset	Flipping	Graph	Weighted F1	Accuracy
BUHMAP	no	base	$87.05{\%}_{\pm 1.79}$	$86.75_{\pm 1.80}$
BUHMAP	yes	base	$90.64{\%}_{\pm 1.12}$	$90.45_{\pm 1.25}$
LSE_GFE	no	base	$79.17{\%}_{\pm 1.45}$	$79.16_{\pm 1.50}$
LSE_GFE	yes	base	$80.38{\%}_{\pm 0.95}$	$80.37_{\pm 0.93}$

**Table 8:** Ablation study for graph topology.

Dataset	Graph	Weighted F1	Accuracy
BUHMAP	base-graph	$90.64{\%}_{\pm 1.12}$	$90.45_{\pm 1.25}$
BUHMAP	empty-graph	$91.48{\%}_{\pm 1.17}$	$91.36_{\pm 1.23}$
LSE_GFE	base-graph	$80.38{\%}_{\pm 0.95}$	$0.8037_{\pm 0.93}$
LSE_GFE	empty-graph	$79.15{\%}_{\pm 0.66}$	$0.7905_{\pm 0.0062}$

**Table 9:** Ablation study with one single scale in both spatial and temporal dimensions.

Dataset	Graph	(SS,TS)	Weighted F1	Accuracy	Parameters
BUHMAP	base-graph	(8,8)	$90.64{\%}_{\pm 1.12}$	$90.45_{\pm 1.25}$	6527396
BUHMAP	base-graph	(1,1)	$92.75{\%}_{\pm 1.60}$	$92.66_{\pm 1.67}$	2159676
BUHMAP	empty-graph	(8,8)	$91.48{\%}_{\pm 1.17}$	$91.36_{\pm 1.23}$	6527396
BUHMAP	empty-graph	(1,1)	$93.21{\%}_{\pm 0.82}$	$93.09_{\pm 0.86}$	2159676
LSE_GFE	base-graph	(8,8)	$80.38{\%}_{\pm 0.95}$	$80.37_{\pm 0.93}$	6525856
LSE_GFE	base-graph	(1,1)	$79.99{\%}_{\pm 0.92}$	$79.86_{\pm 0.93}$	2158136
LSE_GFE	empty-graph	(8,8)	$79.15{\%}_{\pm 0.66}$	$79.05_{\pm 0.62}$	6525856
LSE_GFE	empty-graph	(1,1)	$79.93{\%}_{\pm 0.61}$	$79.81_{\pm 0.64}$	2158136

**Table 10:** Ablation study on duration and FPS. Empty-graph, horizontal flipping and one single scale in both spatial and temporal dimensions.

Dataset	Duration	FPS	Weighted F1	Accuracy
BUHMAP	4 s	30	$89.87{\%}_{\pm 0.84}$	$89.0_{\pm 0.93}$
BUHMAP	4 s	15	$93.21{\%}_{\pm 0.82}$	$93.09_{\pm 0.86}$
BUHMAP	2 s	30	$92.83{\%}_{\pm 0.96}$	$92.75_{\pm 1.01}$
BUHMAP	2 s	15	$92.68{\%}_{\pm 0.82}$	$92.57_{\pm 0.89}$
LSE_GFE	4 s	30	$68.67{\%}_{\pm 1.89}$	$68.23_{\pm 2.00}$
LSE_GFE	4 s	15	$70.42{\%}_{\pm 1.43}$	$70.20_{\pm 1.43}$
LSE_GFE	2 s	50	$79.93{\%}_{\pm 0.61}$	$79.81_{\pm 0.64}$
LSE_GFE	2 s	20	$80.70{\%}_{\pm 0.97}$	$80.45_{\pm 1.01}$

**Table 11:** Comparison of performance over BUHMAP against other published works.

System	Classes	Eval. Method	Accuracy	F1-Score
[41]	All	LOSO CV	76.98%	
[42]	2 to 8	LOSO CV	98.2%	
[43]	1, 5 and 7	Unknown	88.50%	88.87%
[44]	2 a 8	1 person test	67.1%	
[44]	2, 3, 4 and 7	1 person test	95.0%	
[44]	2 a 8	5-fold CV	92.5%	
[44]	2, 3, 4 and 7	5-fold CV	96.6%	
[44]	2, 3, 4 and 7	2 persons test	91.6%	
Ours (best)	All	LOSO CV	93.09%	93.21%

## Data Availability

All the data and code needed to reproduce the experiments of this work can be obtained from https://github.com/mporta-gtm/GrammaticalFacialExpressions (accessed on 1 May 2022).
